# A cross-sectional study of psychache and coping strategies and therapeutic approaches among Palestinian university students during the Gaza Israeli war

**DOI:** 10.1038/s41598-025-03998-2

**Published:** 2025-07-01

**Authors:** Suheir S. Sabbah, Zaid Yacoub, Hamzeh Yacoub, Rita Yacoub, Ronald R. Holden

**Affiliations:** 1 Department of Psychology, Al Quds University, Abu Dis, Jerusalem, Palestine; 2 Faculty of Medicine, Al Quds University, Abu Dis, Jerusalem, Palestine; 3 Department of Psychology, Queen’s University, Kingston, ON Canada

**Keywords:** Psychache, Palestinian students, Gaza 2023 war, Coping strategies, Mental health intervention, University students, Psychology, Human behaviour, Psychology and behaviour

## Abstract

The prolonged Gaza-Israeli war has profoundly impacted the psychological well-being of Palestinian university students, manifesting in elevated levels of psychache—an intense psychological pain marked by despair, humiliation, and helplessness. Despite its established link to suicidal ideation, the relationship between psychache and coping strategies during wartime remains underexplored. This study aims to assess the prevalence and severity of psychache among Palestinian university students in the West Bank during the Gaza-Israeli war, and to identify the coping strategies employed. A cross-sectional study was conducted with 556 participants from various universities in the West Bank using convenience sampling method. Data were collected via online questionnaires, including the Psychache Scale, Orbach and Mikulincer Mental Pain Scale, and Coping Strategies Questionnaire. Descriptive statistics, t-tests, and ANOVA were used to analyze the data. The findings revealed that 10.1 to 13.5% of students experienced high psychache levels, while 38.8 to 41.2% displayed moderate levels. Factors such as marital status and study year were significantly related to psychache levels. The most prevalent coping strategy was diversion followed by reinterpreting, with catastrophizing being the least used. Psychache was positively correlated with maladaptive coping strategies, particularly catastrophizing (*r* = 0.69, *p* < 0.01). The study underscores the critical need for targeted mental health interventions among university students during war situations. Enhanced coping strategies and mental health support tailored to students’ demographics and psychosocial context are essential for mitigating the impact of war on psychological well-being.

## Introduction

Psychological well-being is profoundly influenced by an individual’s sense of safety and stability. According to Maslow’s Hierarchy of Needs, safety and security form the second level of human needs, ranked just above physiological necessities^1^. When these needs are threatened, particularly in situations of war and conflict, individuals face an increased risk of developing psychological distress, including anxiety, depression, and psychache^2,3^. During recent years, Palestinians in the West Bank and Gaza have experienced numerous significant military incursions, resulting in thousands of deaths, injuries, and disabilities^4,5^. Each day, the number of civilians surviving with traumatic psychological disorders increases. Indeed, Palestinians have had to experience all types of fears concerning Israeli aggressions expanding into the West Bank and Gaza, as well as feelings of uncertainty, powerlessness, and distress when faced with images and information of pain and suffering^4^.

Many factors can affect individual persons’ psychological functioning, including financial stress^2^. Of note, prior to October 2023, Palestine was considered an upper middle-income country^6^. Since that time, however, the financial status of the Palestinian authority has deteriorated, resulting in a huge fiscal crisis, according to data from the world bank^7^. It has been reported that approximately 500,000 jobs have been lost for Palestinians since that time, causing many individuals to face financial difficulties^7^. Overall poverty in Palestine was already increasing prior to the 2023 war and in mid-2023, the poverty rate in Gaza strip was nearly twice that of the West Bank^3^. The Palestinians are considered a young nation with 59% of the total population aged between 14 and 65 years and only 3% aged 65 years or greater^8^. Of note, university students represent a population of particular vulnerability for developing mental health problems because these students are transitioning from adolescence to adulthood, facing new academic pressures and challenges, and experiencing economic instability, making them more prone to developing mental health problems including mental pain (i.e., psychache)^9^. These challenges may affect their academic performance and require them to modify their daily routines^10^. In the context of Palestinian students living in war zones, the constant violence and displacement threats greatly disrupt students’ sense of safety, which can lead to elevated stress, anxiety, depression levels, and other psychological issues^11^.

A study conducted by Elbedour et al. with 229 Palestinian adolescents living in Gaza found that 68.9% had developed PTSD, 40.0% reported moderate or severe depression, 94.9% experienced severe anxiety, and 69.9% showed suboptimal coping responses in the wake of the second intifada (uprising). Adolescents diagnosed with PTSD tended to be those who reported the highest levels of depression, anxiety, and positive reappraisal coping, and the lowest levels of seeking guidance and supportive coping^12^.

Psychache (i.e., unbearable mental pain) has been extensively studied in relation to suicidal behavior^13^. Research has consistently shown that it is a major factor in the understanding of suicide^14^. A systematic review by Cavanagh and colleagues of psychological autopsy studies of suicides revealed that approximately 90% of persons who had died by suicide in Europe and North America had been diagnosed with a mental disorder and approximately 60% had a mood disorder diagnosis^15^. In a study conducted on veterans with suicidal ideation and/or behavior, in which the Mee-Bunney Psychological Pain Scale (MBP) and the Columbia-Suicide Severity Rating Scale were used, it was reported that 42.1% of suicidal patients had high levels of psychological pain. The researchers concluded that the MBP has the highest predictive value for suicidality in comparison to the Beck Depression Inventory scale, the Beck Hopelessness Scale, and the Barratt Impulsiveness Scale^16^. The literature has also shown that psychache is a stronger predictor of suicidality than PTSD, particularly in populations exposed to chronic conflict and war. A meta-analysis by Verrocchio et al.^17^ found that psychache mediates the relationship between trauma exposure and suicidal ideation, suggesting that it serves as a more direct mechanism than PTSD in explaining suicidal behavior.

The term “psychache” was introduced by Shneidman who defined it as “the pain which is characterized by intense feelings of shame, humiliation, hurt, anguish, despair, loneliness, fear, and dread”^18^. It is associated with feelings of failure, abandonment, emotional overwhelmingness, emptiness, and a belief that the pain is irreversible^19^. It is distinct from physical pain because it is non-localized and is not associated with the destruction of body tissue^20^. An extensive contribution in linking psychological pain and suicide intent has been provided in Shneidman’s Cubic model of suicide. Shneidman presumed that psychache is a major predisposing factor for suicidal thoughts and processes. His theory considered suicide as a direct result of unbearable and intolerable psychological pain^21^. Shneidman postulated that psychache was the core component of the suicidal process, explaining suicidal ideation, attempts, and death by suicide^13^.

First introduced by Lazarus, coping is defined as “cognitive and behavioral efforts to manage specific external or internal demands that are appraised as taxing or exceeding the resources of the person”^22^. Psychological stress is the result of the failure of coping, when environmental demands exceed a person’s resources (i.e., coping mechanisms)^22,23^. Measurement of coping has included various methods and questionnaires; the one used here is the Coping Strategies questionnaire (CSQ) which was modified for the use of patients with chronic psychological and physiological pain to measure cognitive and behavioral coping strategies^24^.

Associations among coping, psychache, and mental health outcomes have been examined but not during war time. In a study of a Canadian armed forces sample, D’Agata et al. reported that avoidant coping was significantly related to psychache (r = 0.68) and to various negative health outcomes including Post-Traumatic Stress Disorder (rs = 0.29 to 0.67), and that psychache significantly mediated each of the links between avoidant coping and negative health outcomes^25^.

Despite the extensively reported data of the literature about Post-Traumatic Stress Disorder (PTSD) which has been the focus in war-related research in Palestine, to date, to the best of our knowledge, no study has examined the relationship between psychache and coping mechanisms among university students during war time^11,12,26^. This cross-sectional study aims to fill this gap by investigating the prevalence of psychache among Palestinian university students during the Gaza-Israeli war and examining the relationship between psychache and different coping strategies. Addressing this important relation is essential for developing evidence-based mental health programs tailored to the unique experiences of students living in war zones.

Accordingly, based on the cited previous research, our study assessed the prevalence of psychache during the Gaza-Israeli war among Palestinian University students living in the West Bank using the Psychache Scale as well as the Orbach and Mikulincer Mental Pain Scale^27,28^.

Hypotheses:

### H1

Higher levels of psychache will be significantly associated with higher levels of maladaptive coping strategies (e.g., catastrophizing) among university students in the West Bank.

### H2

Higher levels of psychache will be significantly associated with lower levels of adaptive coping strategies (e.g., reinterpretation, cognitive coping) among university students in the West Bank.

### H3

Demographic factors (e.g., academic year, marital status, field of study) will significantly relate to psychache levels among university students in the West Bank.

### H4

Therapeutic approaches will significantly relate to psychache levels.

## Method

### Participants

The study included 556 students meeting the inclusion criteria: a Palestinian university student who is over 18 years old, and there were no exclusion criteria. Given the psychological toll of war, we specifically selected students from the West Bank to assess psychache and coping strategies during the Gaza-Israeli war. This population was chosen due to their direct exposure to conflict-related stressors, which significantly impact mental health. Participating students were a sample of convenience, and data were collected between June and July 2024 during the Gaza war.

Participants completed demographic information and three questionnaires, using online Google Forms. Two scales assessed psychological pain (i.e., psychache), the Psychache Scale^27^ and the Orbach and Mikulincer Mental Pain Scale^28^ and one scale measured coping strategies, the Coping Strategies Questionnaire^29^. The sample included 399 women (71.8%), and 157 men (28.2%), with 66% of them in bachelor programs and 34% at a higher educational level (master’s and doctoral).

### Materials

Holden et al.’s previously validated 13-item Psychache Scale assessed psychological pain using 9 items scored on 5-point Likert ratings from 1 (Never do that) to 5 (Always do that), and 4 items scored on 5-point Likert ratings from 1 (Strongly disagree) to 5 (Strongly agree)^27^.

The Orbach and Mikulincer Mental Pain Scale^28^ is a validated and a reliable measure used in various research. It is an 8-item questionnaire divided into three subscales: Irreversibility (IRR) (2 items), Emotional Flooding (EF) (3 items), and Narcissistic Wounds (NW) (3 items). The total scale score was used as a corroborating measure to the Psychache Scale.

The Coping Strategies Questionnaire (CSQ)^29^ consists of 23 items measuring four coping strategies: Catastrophizing (6 items), this strategy is defined as the propensity to overestimate both the probability and severity of potential negative outcomes associated with symptoms^30^; Diversion (6 items) which is considered as an avoidance coping strategy^31^; Reinterpreting (6 items), that is re-evaluating a stressful situation to perceive it more positively^32^; and Cognitive Coping (5 items) which is efforts to alter one’s perception or understanding of a situation^33^.

A (yes or no) set of questions was also included to evaluate therapeutics used by participants to mitigate the psychological pain they were experiencing. These included options such as “Training and yoga” and “Using substances”. Descriptive statistics and internal consistency reliabilities of the scale scores are shown in (Table 1).

**Table 1:** Descriptive statistics and coefficient alpha reliabilities of study scales.

Scale	Possible range	Observed range	Coefficient α
Psychache Scale	13–65	13–65	0.94
Orbach and Mikulincer Mental Pain Scale	8–40	8–40	0.96
Irreversibility	2–10	2–10	0.93
Emotional flooding	3–15	3–15	0.93
Narcissistic wounds	3–15	3–15	0.95
Coping Strategies Questionnaire	23–115	23–115	0.95
Catastrophizing	6–30	6–30	0.94
Diversion	6–30	6–30	0.96
Reinterpreting	6–30	6–30	0.95
Cognitive coping	5–25	5–25	0.96

### Justification for measurement tools

The Psychache Scale was selected due to its well-established validity in measuring psychological pain. Given that psychache is the center of the study’s focus, this scale ensures precise quantification of the construct.

The Orbach and Mikulincer Mental Pain Scale was included as a complementary measure to validate psychache levels, to increase reliability, and to explore its specific dimensions (irreversibility, emotional flooding, and narcissistic wounds).

The Coping Strategies Questionnaire (CSQ) was chosen because it differentiates between adaptive and maladaptive coping mechanisms, providing insights into how Palestinian university students manage psychological distress.

The Therapeutic Approaches Section was added to reflect the variety of mental health management strategies used by students. These approaches range from religious practices such as prayer and worship, which play a central role in Palestinian culture, to psychotherapy and pharmacological interventions. Including therapeutic methods in the study enhances the understanding of how students manage psychache and allows for culturally relevant mental health interventions to be designed.

By using these three scales and incorporating therapeutic approaches, this study ensures a comprehensive assessment of psychological pain, its manifestations, and the coping mechanisms employed by university students in a war-affected region.

### Design and procedure

The goal of this cross-sectional study was to shed light on the prevalence of psychache (i.e., psychological pain) among universities students in Palestine. A University Institutional Review Board approved the study, and participants provided informed consent prior to participation. Scales were translated into Arabic and an expert reviewed the translations to ensure accuracy. Further, two psychologists reviewed the material to ensure face validity. A pilot study with 41 respondents indicated that all items within the scales had strong item-total correlations (*p* < 0.05) and, thus, no items were deleted from the scales. Data collection was conducted using Google Forms and distributed through various university groups.

### Sociodemographic and characteristics of the participants

The sample of 556 students came from various universities in the West Bank, Palestine with approximately 72% (N = 399) of the participants being women. Furthermore, most undergraduates were fourth-year students (N = 107; 29.2%), followed by third-year students (N = 96; 26.2%), second-year students (N = 92; 25.1%), first-year students (N = 43; 11.7%), fifth-year students (N = 18; 4.9%), and sixth-year students (N = 11; 3.0%). Regarding study programs, more than half the sample (N = 285; 51.3%) were studying in science faculties. Additionally, 66% (N = 367) were pursuing bachelor’s and doctoral degrees, while approximately 70% (N = 388) lived in areas under Palestinian government control (Table 1). The participants’ marital status varied, with the majority being single (N = 310; 55.8%), followed by being married (N = 189; 34%), and either divorced or widowed (N = 57; 10.3%) (Table 2).

**Table 2:** Demographic characteristics of participants.

Characteristic	Frequency (N)	Percentage (%)
Gender		
Men	157 (28.2%)	28.2
Women	399 (71.8%)	71.8
Year of study among undergraduates		
First year	43 (11.7%)	12.4
Second year	92 (25.1%)	33.8
Third year	96 (26.2%)	27.2
Fourth year	107 (29.2%)	20.7
Fifth year	18 (4.9%)	3.4
Sixth year	11 (3.0%)	2.5
Students’ Specializations		
Science	285 (51.3%)	51.3
Humanities and Social Sciences	271 (48.7%)	48.7
Study level		
Bachelor	367 (66.0%)	66.0
Higher education level (master’s and doctoral)	189 (34.0%)	34.0
Residency		
Under Palestinian control	388 (69.8%)	69.8
Under Israeli control	168 (30.2%)	30.2
Marital status		
Single	310 (55.8%)	55.8
Married	189 (34.0%)	34.0
Divorced	50 (9.0%)	9.0
Widow/er	7 (1.3%)	1.3

### Statistical analysis

**Fig. 1:**
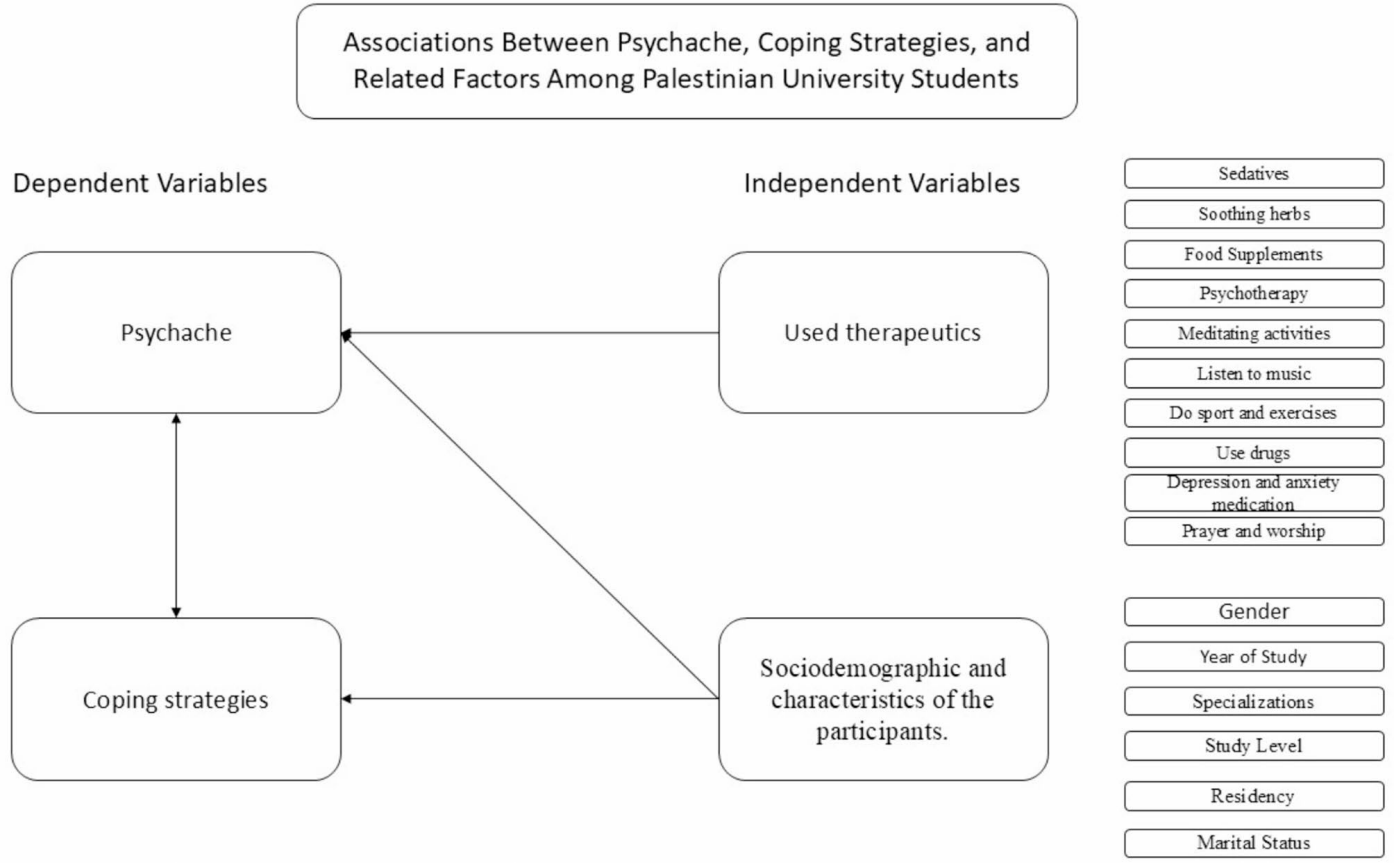
Diagrammatic illustration of the various study factors.

The Statistical Package for Social Sciences (SPSS version 22) was used in data analysis. Answering all items was a requirement for completing the questionnaires, so no missing data were present. First, descriptive analyses were undertaken, generating data as frequencies and percentages for categorical variables. Subsequently, analyses involved t-tests, analyses of variance, and correlations with statistical significance defined as *p* < 0.05. Figure 1 offers a visual depiction of this research showing relationships between the various factors and their potential effects.

## Results

### Psychache level among the sample

In this study, because the Psychache Scale and Orbach and Mikulincer Mental Pain Scale have no generally established normative data, psychache scores were classified into low, moderate, and high categories based on interval scaling principles^34^. For the Psychache Scale the total range was divided into three nearly equal intervals: low (13 to 29), moderate (30 to 46), and high (47 to 65). Similarly, the Orbach and Mikulincer Mental Pain Scale was also divided into three intervals, resulting in low (8 to 18), moderate (19 to 29), and high (30 to 40) categories. This method, grounded in equal interval scaling, ensures accurate identification of distress levels. Psychache levels varied among students, with low pain levels being the most prevalent in the sample (48.7% using the Psychache Scale and 47.7% using the Orbach and Mikulincer Mental Pain Scale) (Table 3).

**Table 3:** Psychache level in the sample.

Scale	Mean	*SD*	Participants with high pain	Participants with moderate pain	Participants with low pain
Psychache Scale	31.68	10.91	56 (10.1%)	229 (41.2%)	271 (48.7%)
Orbach and Mikulincer Mental Pain Scale	20.43	7.05	75 (13.5%)	216 (38.8%)	265 (47.7%)

Significant values are in [italics].

Psychache levels varied significantly as a function of several factors, including students’ specializations, year of study, study level, and marital status (Table 4). However, gender and residency place did not relate to the level of psychache significantly (*p* > 0.05). Marital status significantly related to psychache levels, with single students exhibiting higher mean levels than married students, and divorced/widowed students displaying higher mean levels than either single or married students. The year of study for undergraduates significantly related to the level of psychache, with sixth-year students having the lowest level of psychological pain, followed by first-year students, while third and fifth-year students had the highest levels of psychological pain. Additionally, the Orbach and Mikulincer Mental Pain Scale indicated that students pursuing humanities and social sciences majors reported higher levels of psychache. Higher education degree students experienced more psychological pain compared to those pursuing their bachelor’s degrees. (Table 4).

**Table 4:** Demographics and the level of psychache among the sample.

Characteristics	Scale of psychache			Orbach and Mikulincer Mental Pain Scale		
	M	*SD*	t/F value	M	*SD*	t/F value
Gender			− 0.95			− 0.10
Men	30.97	11.60		20.39	7.60	
Women	31.96	10.63		20.45	6.83	
Students’ Specializations			0.80			− 2.28**
Scientific	32.06	12.55		19.74	6.96	
Humanities and Social Sciences	31.32	9.10		21.10	7.08	
Year of study among undergraduates			3.24**			5.73**
First year	30.10	12.93		18.56	7.30	
Second year	30.18	10.54		18.88	6.36	
Third year	34.16	12.31		22.65	7.25	
Fourth year	32.64	11.91		18.90	6.65	
Fifth year	33.50	10.31		19.33	5.86	
Sixth year	22.36	6.39		16.20	6.00	
Study level			0.53			− 3.14**
Bachelor	31.85	11.78		19.77	6.95	
Higher education level (Master’ and PhD)	31.33	9.02		21.73	7.08	
Residency			0.61			− 1.80
Under Palestinian control	31.88	11.54		20.14	7.19	
Under Israeli control	31.24	9.11		21.36	6.44	
Marital status			2.83**			2.88**
Single	32.73	12.16		20.00	6.97	
Married	29.89	8.95		20.39	7.06	
Divorced	31.50	8.39		23.02	7.26	
Widow/er	34.57	12.39		22.57	5.41	

**p value < 0.05.

Significant values are in [italics].

### Coping strategies within the sample

This study measured four coping strategies within the sample. We found that the most used strategy was diversion with catastrophizing being the least used strategy (Table 5). Several statistically significant characteristics were associated with the use of different coping strategies. Sixth-year undergraduate students showed the least tendency to use the catastrophizing coping strategy. The diversion strategy was used the most among women and widow/er students. Third-year undergraduate students and master’s and doctoral students had the highest use of the reinterpreting strategy. Cognitive coping was highest among women and widow/er students (Table 6).

**Table 5:** Mean and standard deviation of the different coping strategies used.

Coping Strategy	Mean	*SD*
Catastrophizing	13.82	5.89
Diversion	19.34	6.36
Reinterpreting	16.52	5.92
Cognitive Coping	15.88	5.32

Significant values are in [italics].

**Table 6:** Characteristics for using different coping strategies.

Coping strategy	Characteristics	M	*SD*	t/F Value
Catastrophizing	Year of study among undergraduates			3.30**
	First year	12.56	6.40	
	Second year	12.90	5.71	
	Third year	15.36	6.32	
	Fourth year	13.74	5.82	
	Fifth year	13.40	6.70	
	Sixth year	9.27	4.70	
Diversion	Gender			− 5.83**
	Men	16.90	6.35	
	Women	20.30	6.11	
	Marital status			15.80**
	Single	16.44	5.80	
	Married	16.43	6.14	
	Divorce	16.82	5.83	
	Widow/er	20.00	5.65	
Reinterpreting	Year of study among undergraduates			2.80**
	First year	15.42	6.41	
	Second year	15.90	6.33	
	Third year	17.51	5.70	
	Fourth year	15.87	5.16	
	Fifth year	15.67	4.81	
	Sixth year	11.50	5.63	
	Study level			− 2.27**
	Bachelor	16.11	5.83	
	Higher education level (Master’s and doctoral)	17.31	6.04	
Cognitive Coping	Gender			− 2.80**
	Men	14.89	5.60	
	Women	16.28	5.16	
	Marital status			8.01**
	Single	16.73	5.18	
	Married	14.64	5.43	
	Divorced	14.84	4.75	
	Widow/er	19.43	4.50	

**p value < 0.05.

Significant values are in [italics].

### Therapeutic approaches

University students employed a range of therapeutic approaches to manage their psychological pain ranging from participating in regular physical exercises to resorting to substance use. Among all approaches, prayer and worship were the most used methods, followed by the use of soothing herbs. Students tended to use conventional therapeutic approaches. Higher levels of psychache were observed among students who used soothing herbs, psychotherapy, and those who listened to music. Conversely, students who did engage in the use of sedatives, food supplements, sports and exercise, substances use, medications for depression and anxiety, or prayer and worship experienced lower levels of psychological pain (Table 7).

**Table 7:** Psychache therapeutic approaches in the sample.

Therapeutic approach		Frequency	Psychache level	
			Psychache scale	Orbach and Mikulincer Mental Pain Scale
Sedatives	Yes	229 (41.2%)	30.80	18.52
	No	327 (58.8%)	32.30	19.68
Soothing herbs	Yes	318 (57.2%)	31.90	20.99
	No	238 (42.8%)	31.39	19.70
Food Supplements	Yes	236 (42.4%)	31.49	19.98
	No	320 (57.6%)	31.82	20.78
Psychotherapy	Yes	190 (34.2%)	31.77	21.28
	No	366 (65.8%)	31.63	20.00
Meditating activities	Yes	295 (53.1%)	31.30	19.71
	No	261 (46.9%)	32.11	20.14
Listen to music	Yes	329 (59.2%)	31.93	20.50
	No	227 (40.8%)	31.32	20.35
Do sport and exercises	Yes	296 (53.2%)	30.91	19.92
	No	260 (46.8%)	32.57	21.03
Substance use	Yes	107 (19.2%)	30.17	19.01
	No	449 (80.8%)	32.04	20.30
Depression and anxiety medication	Yes	153 (27.5%)	30.64	20.67
	No	403 (72.5%)	32.08	21.35
Prayer and worship	Yes	429 (77.2%)	31.46	19.67
	No	127 (22.8%)	32.42	23.05

### The relationship between psychache and coping strategies

The Psychache Scale had a strong positive correlation with the Orbach and Mikulincer Mental Pain Scale (*r* = 0.71, *p* < 0.01) and the catastrophizing coping strategy (*r* = 0.69, *p* < 0.01), and positively correlated with the other coping strategies at *p* < 0.01. The Orbach and Mikulincer Mental Pain Scale was positively correlated with all coping strategies at *p* < 0.01, with the strongest correlation being with the catastrophizing coping strategy (*r* = 0.71, *p* < 0.01). Positive correlations were observed among the coping strategies themselves, with the strongest correlation between the reinterpretation strategy and the cognitive coping strategy (*r* = 0.82, *p* < 0.01) (Table 8).

**Table 8:** Correlations between the study variables.

Variables	Psychache scale	Orbach and Mikulincer Mental Pain	Catastrophizing	Diversion	Reinterpreting	Cognitive coping
Psychache Scale						
Orbach and Mikulincer Mental Pain Scale	0.71**					
Catastrophizing	0.69**	0.71**				
Diversion	0.32**	026**	0.29**			
Reinterpreting	0.38**	0.46**	0.44**	0.55**		
cognitive coping	0.26**	0.23**	0.20**	0.69**	0.82**	

**p value < 0.05.

## Discussion

Psychache, also referred to as psychological or mental pain^35^ has been extensively studied by researchers, particularly in relation to its connections with suicidal ideation and attempts, depression, and anxiety disorders. However, the impact of war on psychache has not been widely investigated. To the best of our knowledge, this study is one of the first globally to explore the association between war and psychache in a population, and the first to assess psychache levels among Palestinians in the West Bank during the Israeli war in Gaza. West Bank citizens and Gazans are deeply connected by familial, cultural, and sociopolitical ties, for which anxiety, depression, and psychache are significantly anticipated to occur among the West Bank population, especially among the undergraduates who are more prone to mental disorders in comparison to the general population^36^.

The results of the study revealed that students at West Bank universities have an overall low psychache level (Mean = 31.68 out of 65, *SD* = 10.91 on the Psychache Scale; Mean = 20.43 out of 40, *SD* = 7.05 on Orbach and Mikulincer Mental Pain Scale). Notably, 10.1% and 13.5% of the sample suffer from high psychache levels, while 41.2% and 38.8% showed moderate levels on the Psychache Scale and the Orbach and Mikulincer Mental Pain Scale, respectively. Despite the overall low level of psychache among university students, the significant proportion of students with high and moderate levels suggests a potential risk of suicide as indicated by Orbach and Verrhocchio et al.^17,19^ Several studies have demonstrated psychache as a risk factor for suicide. Shneidman highlighted that psychache is essential for suicide to occur, emphasizing its greater significance than depression in the decision-making process, as he stated that depression alone does not lead to suicide, unlike psychache^13^. Alongside this, a prospective study has established that the change in psychache over two years was the most critical factor (rather than depression or hopelessness) in predicting suicidal ideations^37^. Additionally, Siau et al.^38^ have found a positive interaction between psychache, hopelessness, and suicidal ideation among Chinese undergraduates. Studies have interpreted suicidal ideation within individuals as a way of escaping unbearable pain associated with unfulfilled psychological needs such as security^39,40^.

Compared to studies conducted in peaceful regions, where psychache level was lower than that observed in our study^27,41^, our findings suggest that the ongoing conflict may exacerbate mental pain among students. Additionally, although the overall level of psychache among West Bank university students was low, the significant proportion of high and moderate levels are concerning. This is consistent with the broader literature on mental health in conflict zones, which suggests that individuals in war areas are vulnerable to neuropsychiatric conditions such as depression, PTSD, and anxiety^2^.

Demographic characteristics showed variations regarding psychache levels, except for sex and place of residency, where the results were not statistically significant (*p* > 0.05). This finding does not match the results of a study conducted on Hakka elderly adults, which revealed that elderly women had higher pain levels than men^42^. Our finding suggests that psychache is a universal phenomenon in the university student population, especially during wars, where it is independent of sex and not influenced by gender-related factors. On the other hand, the year of study had a significant impact on pain levels within undergraduate students, with third- and fifth-year students exhibiting the highest levels, while first- and sixth-year students had the lowest levels. This finding contrasts with previous studies which demonstrated that first year students had more psychological distress and lower resilience than students with more years in university^43,44^. This may be attributed to the effect of war on education. The emergence of online learning has prevented first-year students from entering the university campus and experiencing the challenges of a new environment. Moreover, students pursuing humanities and social sciences majors showed greater levels of pain than other students. The latter finding can be interpreted in two ways; first, humanity and social sciences students often engage in critical thinking, self-reflection, and discussions on human sufferings, as well as the injustice and existential issues of the world, in addition to analyzing ethical dilemmas in some of these fields. Hence, their study may place them at deeper emotional engagement with topics such as trauma, wars, inequity, and psychological challenges, which makes them more vulnerable to psychache. Additionally, students in social sciences usually face less structured career paths compared to those in the scientific fields. This challenge is particularly notable in the Palestinian environment, especially during the war, because career opportunities have substantially declined.

Marital status also related to psychache level; students who were not in a relationship (single, divorced, or widow) had higher levels compared to married students. This finding aligns with some studies that have shown relationship status is associated with mental well-being. A Finnish cohort study followed up a sample at different age phases during life, for persons being in varied relationships, and concluded that single and divorced/ widowed status were risk factors for depressive symptoms and worse mental well-being compared to those who were in a partnered relationship^45^.

In addition, our study investigated the different feelings among Palestinians who reside in areas controlled by the Palestinian authority and those who reside in areas under Israeli control, i.e., students who live in Palestinian areas that were occupied in 1948, with both groups registered in West Bank universities. Results revealed that both groups suffered from the same level of psychache.

### Coping strategies among the sample

Yeşiloǧlu et al. demonstrated that coping skills significantly mitigate psychache^46^, As well, other studies have shown that coping strategies play the most important role in helping individual’s mental health during conflicts and wars^23,47^, a finding supported by our assessment of four coping strategies: catastrophizing, diversion, reinterpreting, and cognitive coping. Among these, diversion emerged as the most prevalent (Mean = 19.34), followed by reinterpreting and cognitive coping, with catastrophizing being the least utilized (Mean = 13.82). Given that catastrophizing is widely regarded as a maladaptive coping strategy^48^, its association with poorer health-related quality of life is well-documented^49^.

Although different studies have demonstrated that gender does not relate to the utilization of particular coping strategies in the population^50,51^, our study showed women tend to use the diversion and cognitive coping strategies more than men. The high use of diversion by women in our study (involving engaging in distractions to shift away from stress) is consistent with the result of a study by Sinha and Latha, which found that female students commonly use emotional-focused coping strategies^52^. In the Palestinian society, women have additional family responsibilities, making diversion a potential tool to temporarily escape emotional distress. Additionally, diversion includes thinking about pleasant experiences or enjoyable activities, which might provide more comfort to women, strengthening their ability to focus on society and family roles. Moreover, cognitive strategies enhance self-encouragement “I can handle it” and reframe pain as a challenge. The Palestinian woman is expected to maintain responsibilities and persevere through hardship, thus, reframing stress as a challenge, and minimizing emotional distress “I just go on as nothing happened” may reflect this anticipated endurance. Similar reasoning may also apply to widow/er students who experience intense challenges due to personal loss and societal expectations.

As well, this study found a higher propensity for catastrophizing among third-year undergraduate students, as compared to other students. This pattern suggests that the elevated stress associated with more demanding academic years may predispose students to adopt less adaptive coping strategies. In contrast, individuals who had experienced significant personal losses, such as divorce or the death of a significant other, demonstrated a greater tendency to use diversion and cognitive coping strategies. These findings indicate that the challenges associated with such life experiences, alongside the greater age of these individuals, may foster the development of more adaptive coping mechanisms. This trend highlights the potential influence of life experience and age on the selection of coping strategies, warranting further investigation.

A systematic review by Hamadeh et al. on coping strategies in conflict zones among individuals from Middle Eastern Arab countries highlighted that individuals in war zones often rely on social and family support, faith, and resilience as key coping strategies. These findings align with our study, where diversion and cognitive coping were prevalent^53^. On the other hand, in comparison to studies conducted in peaceful regions, such as the study by Alkhawaldeh et al. in Oman, our sample exhibited a higher reliance on diversion and cognitive coping, which are often associated with adaptive coping mechanisms. Alkhawaldeh et al.^54^ found that university students in Oman primarily used problem-solving and social support as coping strategies. This may reflect a discrepancy in the coping strategies mostly utilized in situations of trauma as compared to general peace.

### Therapeutic approaches

Praying and maintaining a close relationship with religion are widely recognized methods for stress relief^55^. Given that the Palestinian community is known for its strong religious values^56^, most students in the sample reported using prayer as their primary therapeutic approach to manage their psychache. This aligns with the cultural and religious foundation of the Palestinian society, where religious practices and worship play a significant role in shaping structure, regulating emotion, and providing a sense of meaning. Additionally, while the majority of Palestinians are Muslims, previous studies have highlighted that Muslims typically turn to the practices of Islam to cope with trauma in times of crisis^57–59^. A previous study detected that religious coping was widely used in a Muslim group of refugees to endure post-traumatic stress (PTS) and discrimination. It ultimately found that religious coping was highly associated with greater levels of Traumatic Growth (TG)^57^. Souca has highlighted that religious support emerged as a significant protective factor for Palestinian women exposed to political violence^60^. This correlation predicts a positive psychological change in the religious society after trauma. In contrast, drug use is considered taboo and socially unacceptable, making it the least utilized therapeutic method within our sample. Additionally, medications for depression and anxiety require a doctor’s prescription. Even though seeking psychotherapy has been demonstrated as an important and efficient therapeutic approach in wars and conflicts^61,62^, due to the stigma surrounding mental health and a general reluctance to seek psychotherapy, the study found that the use of antidepressants, anxiety medications, and psychotherapy was relatively uncommon among participants.

### The relationship between psychache and coping strategies

The concept of war alters humans’ mental decisions and reshapes public viewpoints^63,64^, and this may change the population’s coping strategy choice. Our study found psychache levels were highly positively correlated with catastrophizing compared to other coping strategies demonstrating that pain may make students more liable to catastrophize about things and cope negatively towards pain. This finding aligns with our hypothesis that higher levels of psychache are associated with higher levels of maladaptive coping strategy.

Research by Veronese et al. found that despite the high levels of psychological distress among Palestinians under siege in Gaza, resilience played a pivotal role in mitigating the levels of hopelessness, as well as mental distress (depression, anxiety, and stress) among university students^3^. Hence, the relatively low level of psychache and the low rate of catastrophizing coping strategy may suggest that most university students exhibit a degree of resilience. This aligns with the findings of Hawa et al.^65^ who examined the levels of resilience among Palestinian adults in the Gaza strip in relation to the sociodemographic factors. Their results showed that resilience tended to increase with higher educational attainment. Accordingly, university students, as a highly educated subgroup, would be expected to demonstrate greater resilience when exposed to adversity. Additionally, the Palestinian society is known for its strong social ties and religious beliefs. In regard of this, Brewer-Smith and Koenig concluded that spirituality and religion can be powerful sources of hope and may promote resilience^66^. Furthermore, the exposure to war-related stressors and the possibility of facing life-threatening situations can significantly affect the individual’s control. High self-efficacy may empower the individual to confront challenges and adapt resilience^23^. Given these findings, we recommend conducting further research to assess the levels of resilience among university students in the West Bank.

## Conclusion

This study provides important insights about psychache, coping strategies, the choice of therapeutic approaches for the management of psychache, and their relationships with sociodemographic characteristics among Palestinian universities students during the Gaza-Israeli war. Findings emphasize the importance of following up with students’ mental health especially when a war is taking place, and with taking students’ individual factors into consideration such as students’ specialization, education level, and marital status. These findings also highlight the significance of considering these characteristics in the training of students to enhance their coping strategies with psychache so as to promote better mental health.

## Limitations

Our research has potential limitations. First, the study’s measures were translated into Arabic, and despite rigorous back-translation by bilingual experts, the possibility of translation errors remains. Second, reliance on self-report assumes participants’ honesty and accuracy, which may not be the case. Third, the cross-sectional design limits our ability to establish causality between the variables studied and represents a snapshot of what is an evolving situation. Fourth, the therapeutic approaches scale was a limited scale, which may have restricted the generalizability of the finding. To enhance the generalizability of our findings, future research should replicate this study using different samples, diverse data collection methods, longitudinal designs, and a broader scale for the measurement of therapeutic approaches.

### Data source and relevance

The data in this study were collected from 556 Palestinian university students between June and July 2024. Given the dynamic nature of psychological well-being and external factors influencing students’ mental health, this may impact on the relevance of those statistics in the present day.

## Data Availability

The dataset used and/or analyzed during the current study are available from the corresponding author on reasonable request.
